# Early dynamics of the emission of solvated electrons from nanodiamonds in water†

**DOI:** 10.1039/d2nr03919b

**Published:** 2022-11-01

**Authors:** Franziska Buchner, Thorren Kirschbaum, Amélie Venerosy, Hugues Girard, Jean-Charles Arnault, Benjamin Kiendl, Anke Krueger, Karin Larsson, Annika Bande, Tristan Petit, Christoph Merschjann

**Affiliations:** Helmholtz-Zentrum Berlin für Materialien und Energie GmbH Hahn-Meitner-Platz 1 14109 Berlin Germany tristan.petit@helmholtz-berlin.de christoph.merschjann@helmholtz-berlin.de; Institut für Organische Chemie, Julius-Maximilians-Universität Würzburg Am Hubland D-97074 Würzburg Germany; Freie Universität Berlin, FB Mathematik & Informatik, Artificial Intelligence for the Sciences Arnimallee 12 D-14195 Berlin Germany; CEA, LIST, Diamond Sensors Laboratory Bâtiment 451 PC 45 91191 Gif sur Yvette Cedex France; Université Paris-Saclay, CEA, CNRS, NIMBE 91191 Gif sur Yvette Cedex France; Institute of Organic Chemistry, University of Stuttgart, Pfaffenwaldring 55 D-70569 Stuttgart Germany; Uppsala University Lägerhyddsvägen 1 751 21 Uppsala Sweden

## Abstract

Solvated electrons are among the most reductive species in an aqueous environment. Diamond materials have been proposed as a promising source of solvated electrons, but the underlying emission process in water remains elusive so far. Here, we show spectroscopic evidence for the emission of solvated electrons from detonation nanodiamonds upon excitation with both deep ultraviolet (225 nm) and visible (400 nm) light using ultrafast transient absorption. The crucial role of surface termination in the emission process is evidenced by comparing hydrogenated, hydroxylated and carboxylated nanodiamonds. In particular, a transient response that we attribute to solvated electrons is observed on hydrogenated nanodiamonds upon visible light excitation, while it shows a sub-ps recombination due to trap states when excited with deep ultraviolet light. The essential role of surface reconstructions on the nanodiamonds in these processes is proposed based on density functional theory calculations. These results open new perspectives for solar-driven emission of solvated electrons in an aqueous phase using nanodiamonds.

## Abstract 2

Ultrafast transient absorption on nanodiamonds reveals synergistic effects between hydrogen termination and sp^2^-hybridized surface reconstructions, facilitating visible-light driven electron emission.

## Graphical abstract

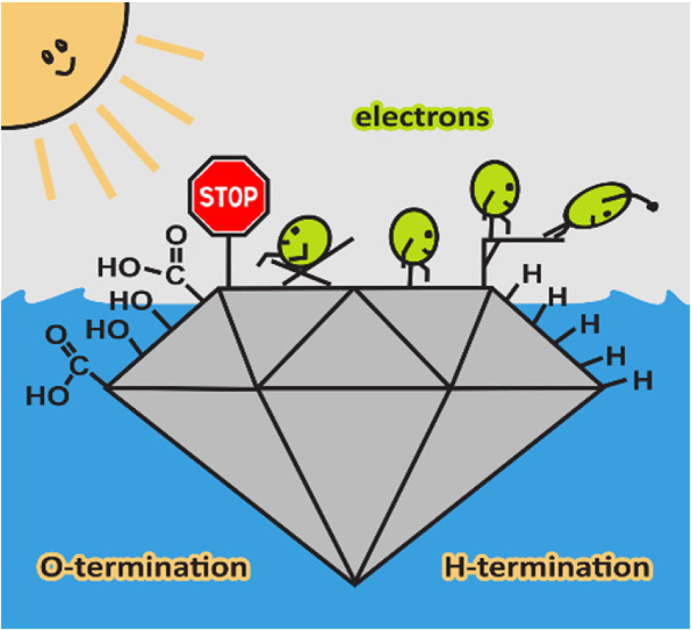

## 1 Introduction

Solvated electrons are highly reductive chemical species involved in many reactions from radiation chemistry to photocatalysis.^1^ Different sources have been considered for the emission of solvated electrons from ionic precursors,^2^ metals,^3–5^ metal oxides^6^ or diamond surfaces.^7^ Diamond materials are particularly interesting due to the negative electron affinity (EA) of the diamond surface when terminated *e.g.* with hydrogen atoms, enabling spontaneous and barrier-free electron emission once electrons are excited to the conduction band.^8^ In particular, diamond has been used to trigger chemical reduction of nitrogen or carbon dioxide directly in the aqueous phase using electrochemical reaction^9,10^ or light excitation.^7,11^ The latter represents a great challenge due to the large bandgap of diamond of 5.5 eV, necessitating the use of deep ultraviolet (DUV) light, which is absent in the solar spectrum.^7^ So far, clear evidence of the light-induced emission of solvated electrons from diamond in water is scarce. Quantitative measurements based on electron scavenging by N_2_O demonstrated an overproduction of solvated electrons in aqueous dispersions of hydrogenated detonation nanodiamonds (NDs) under X-ray irradiation.^12^ An indirect demonstration was provided on boron doped diamond electrodes,^7,13^ NDs^14^ and silver-doped diamond thin films.^15^ In a seminal work, Zhu *et al.* demonstrated that upon DUV light excitation, a transient signal at 633 nm was observed in water above a diamond single crystal which was assigned to solvated electrons.^7^ It was recently completed by a detailed study of transient absorption in the ns–μs range, showing sub-bandgap electron emission for excitation down to 4.5 eV (275 nm).^13^ In these previous studies, the addition of hole or electron scavengers demonstrated that the transient signal was indeed related to solvated electrons. Nevertheless, the time evolution over the early stages (<2 ns) of the electron emission under DUV light remains unexplored to our knowledge. Achieving electron emission with visible (VIS) light excitation instead of DUV light would also be required for a larger significance of diamond in the field of photocatalysis.

A promising strategy to achieve solvated electron emission with VIS light involves the addition of new electronic states within the bandgap of the diamond material that can then be excited with VIS photons above the diamond conduction band minimum (CBM). Several strategies were employed to achieve sub-bandgap absorption, including dye sensitization,^16^ plasmonic coupling,^15^ nanostructuring^17^ and doping.^18,19^ The main challenge is, however, to retain the electron emission properties offered by H-terminated surfaces. Sub-bandgap visible absorption with diamond materials was previously reported in a vacuum^19,20^ but remains to be shown in water. Visible-light reactivity toward CO_2_ photoelectrochemical reduction was recently demonstrated on a hydrogenated nanostructured diamond surface by some of us.^21^ The origin of enhanced reactivity with visible light remains elusive so far. Thus, identifying electron emission processes involved in sub-bandgap (visible) excitation would certainly facilitate the design of more efficient diamond-based electron emitters.

In this study, we have employed ultrafast transient absorption (TA) spectroscopy with sub-ps time resolution to monitor the early stages of solvated electron emission from detonation NDs in water. Detonation NDs consist of a diamond core of 4–5 nm diameter, surrounded by a defective carbon shell,^22^ whose surface chemistry can be controlled by different treatments.^23^ The exact spatial arrangement of detonation NDs in water is still subject to active research, but it is believed to be composed of linear aggregates of polyhedral NDs; their aggregation is driven by short-range electrostatic adsorption, depending on the ND surface charge.^24^ Aqueous dispersions of NDs with hydrogenated (ND-H), carboxylated (ND-COOH) and hydroxylated (ND-OH) surfaces were compared. The impact of excitation photon energy was studied by applying both DUV (225 nm, 5.5 eV) and VIS (400 nm, 3.1 eV) pump laser pulses. All ND surface terminations were found to enable the emission of solvated electrons with DUV light. Furthermore, substantial solvated electron emission was observed for ND-H under visible light. Based on these TA measurements and density functional theory (DFT) calculations, possible mechanisms for the emission of solvated electrons in DUV and VIS regions are proposed.

## 2 Results and discussion

**Fig. 1:**
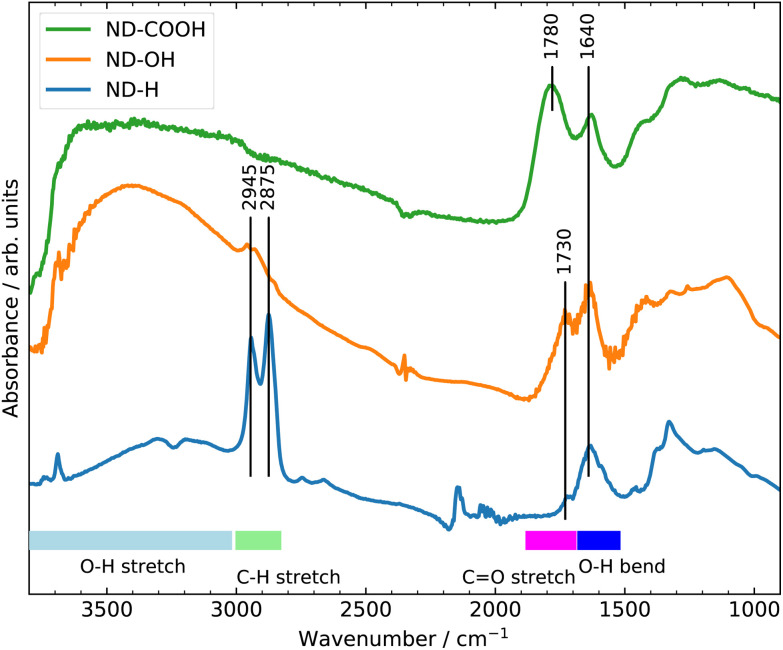
FTIR spectra of ND-COOH, ND-OH and ND-H.

The surface terminations of the three samples were validated by Fourier Transform Infrared (FTIR) spectroscopy as shown in Fig. 1.^25^ The experimental procedures for controlling the surface termination are described in section 4. A strong C=O stretching band at 1780 cm^−1^ related to carboxyl groups is clearly visible on ND-COOH. For ND-OH, the major contribution comes from OH stretching (3200–3600 cm^−1^) and OH bending (1640 cm^−1^) modes from hydroxyl groups, although the contribution from adsorbed water molecules cannot be excluded. The appearance of a broad band at around 1100 cm^−1^ is however more specifically assigned to C–O stretching from hydroxyl groups on NDs. Additionally, the CH_*x*_ groups of primary and secondary hydroxyl functions can be seen at 2800–2900 cm^−1^. A small band at 1730 cm^−1^ related to residual carbonyl is detected but is smaller than the band related to OH bending modes. On ND-H, no C=O band is detected but a double peak (2875 and 2945 cm^−1^), characteristic of the CH_*x*_ groups on NDs is clearly visible. The appearance of a peak at 1330 cm^−1^, previously assigned to C–H scissoring modes,^26^ further confirms the presence of CH_*x*_ groups. In summary, the FTIR confirms the presence of carboxylic acids on ND-COOH and shows the efficient hydrogenation of ND-H after plasma treatment and hydroxylation of ND-OH after the reduction of carboxyl and carbonyl groups.

The ultrafast response of NDs under light excitation is probed by TA experiments with an effective temporal resolution of approximately 150 fs (determined by the pulse durations of the pump and probe pulse, respectively). Further details on the experimental setup and data analysis are available in section 4 and the ESI.† DUV pump pulses were applied to induce the emission of solvated electrons from ND dispersions *via* direct bandgap excitation. In a second set of experiments, VIS light excitation was used to investigate the possibility of solvated electron emission from NDs in water *via* sub-bandgap absorption. Sodium iodide (NaI) solution was used as an ionic source of solvated electrons, for which the solvation dynamics has been extensively studied.^2,27–29^

**Fig. 2:**
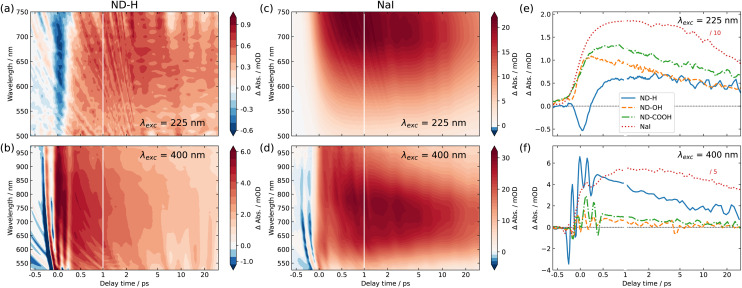
Transient absorption (TA) spectra and traces of nanodiamond aqueous dispersions and NaI solution. (a and b) TA signal from hydrogenated nanodiamond (ND-H) dispersion as a function of probe wavelength and delay time for DUV (a) and VIS (b) excitation. (c and d) The same as (a and b), but for NaI solution. The semilogarithmic time axis and the different absolute signals should be noted. The negative absorbance change around zero delay time in (a) is discussed in the main text, while the multiple sharp variations of the TA signal in the same region in (b and d) are due to cross-phase modulation, induced by the comparably strong VIS pump pulse. (e) Delay-dependent TA traces for NDs, surface-terminated with hydrogen (ND-H), hydroxyl (ND-OH), and carboxyl (ND-COOH) under DUV excitation, averaged over a probe wavelength region of 600–740 nm. For comparison, the TA signal obtained from NaI solution is shown as a dashed red line (the demagnification factor should be noted). (f) The same as (e), but for VIS excitation.

Fig. 2 shows the 2D maps of the time-dependent TA spectra for ND-H (a and b) and NaI (c and d) solutions, under both DUV (a and c) and VIS (b and d) excitation, respectively. The semilogarithmic time axis and the different absolute signals for the different measurements should be noted. In the case of DUV excitation, the probe range is limited to the range 500–750 nm, due to the different probe light generation scheme. The signal obtained for NaI is similar to that obtained in previously reported studies.^2^ For both excitation scenarios, ND-H exhibits a clear positive transient signal at positive delay times. As observed for VIS excitation, the TA signal peaks around 750 nm after about 1 ps, similarly to NaI. The blue shift of the TA maximum throughout the first 1 ps observed for NaI cannot be clearly distinguished for ND-H. The 2D TA traces for other ND samples are available in the ESI (Fig. S2†). In general, the acquired TA spectra are broad and featureless, and substantially weaker than for NaI solutions (Fig. S3†). Nevertheless, the overall TA spectral fingerprint and blue shift over time resembles the NaI signature more than that of the intrinsic electron dynamics in diamond films.^30^ We therefore attribute the TA signal to the signature of solvated electrons. It should be noted that the emission of solvated electrons under ionizing radiation was already demonstrated on the same type of ND-H.^12^ We attempted time-resolved photoemission spectroscopy (PES) to gain further insight into the electronic states of the solvated electrons.^28,29,31^ However, stabilizing a microjet of a ND dispersion in a vacuum for several hours, as needed for the measurements, turned out to be very challenging. Moreover, due to the low concentration of NDs and the limited probing depth of a few nanometres in aqueous solutions, the transient PES signal of solvated electrons was below the detection threshold. Therefore, we concentrate in the following on the temporal profile of the integrated TA response, which provides more information on the dynamics of the electron emission process.

The integrated TA traces over the probe wavelength region of 600–740 nm for ND-H, ND-OH and ND-COOH under DUV excitation are shown in Fig. 2(e). For ND-H, a strong negative TA signal around time zero is observed, which is absent in NaI solution, pure water, and for the other NDs characterized with a similar pump laser intensity (Fig. S1 and S3†). After about 1 ps, a positive signal reaches a maximum and slowly decays over the probed timescale of 30 ps. For ND-OH and ND-COOH, a strong increase of the TA signal occurs in the first 500 fs and it progressively decays similarly to ND-H. Compared to NaI, both build-up and decay kinetics in the first 2 ps appear generally faster for the NDs, with minor differences between the surface terminations. Due to the observed similarities of both their spectral and temporal features, we assign the positive TA signals in the ND dispersions to the emission of solvated electrons upon DUV excitation. The emission of solvated electrons is therefore observed for all surface terminations after DUV excitation above the diamond bandgap.

Slight temporal differences are observed between ND-OH and ND-COOH, which are likely related to the different solvation of the emitted electron in the first picoseconds. Both terminations have indeed opposite zeta-potential, which suggests a different organization of the first hydration shell, hence impacting the electron solvation. In general, the more ordered water hydration structure observed around NDs^32^ may lead to faster kinetics compared to solvated electrons emitted from iodide. The early dynamics was also found to be faster on detonation NDs compared to larger NDs synthesized from High Pressure High Temperature (HPHT) synthesis for carboxylated surfaces (Fig. S4†). Even though solvated electrons are detected from ND-OH or ND-COOH under DUV light, oxidized diamond surfaces have previously shown much lower photocatalytic activity than hydrogenated diamond surfaces.^7^ This can be explained by a surface-dependent diamond-water band alignment as detailed below. Furthermore, an additional ultrafast process occurs only on ND-H during the first picoseconds, which will also be interpreted in the following.

For VIS excitation, an analogous treatment of the 2D TA maps is shown in Fig. 2(f). Compared to the previously used DUV pulses, the VIS pump light contains around 70 times more photons per pulse, which leads to a pronounced and complex coherent artefact also observed for pure water (Fig. S1†). Apart from that, the spectro-temporal behaviour of NaI solution under VIS excitation appears quite similar to that under DUV excitation. For NDs, two notable differences are found compared to DUV excitation. Firstly, no negative TA can be observed for any of the samples, especially not for ND-H. Secondly, a substantial positive TA signal after the initial ultrafast time range is only observable for ND-H. While a TA signal of ≈1 mOD is detected for ND-OH and ND-COOH, its spectral fingerprint is not defined enough to assign it to solvated electrons with confidence (Fig. S3†). Similar to DUV excitation, the kinetics in ND-H is slightly faster than that in the respective NaI solution.

**Fig. 3:**
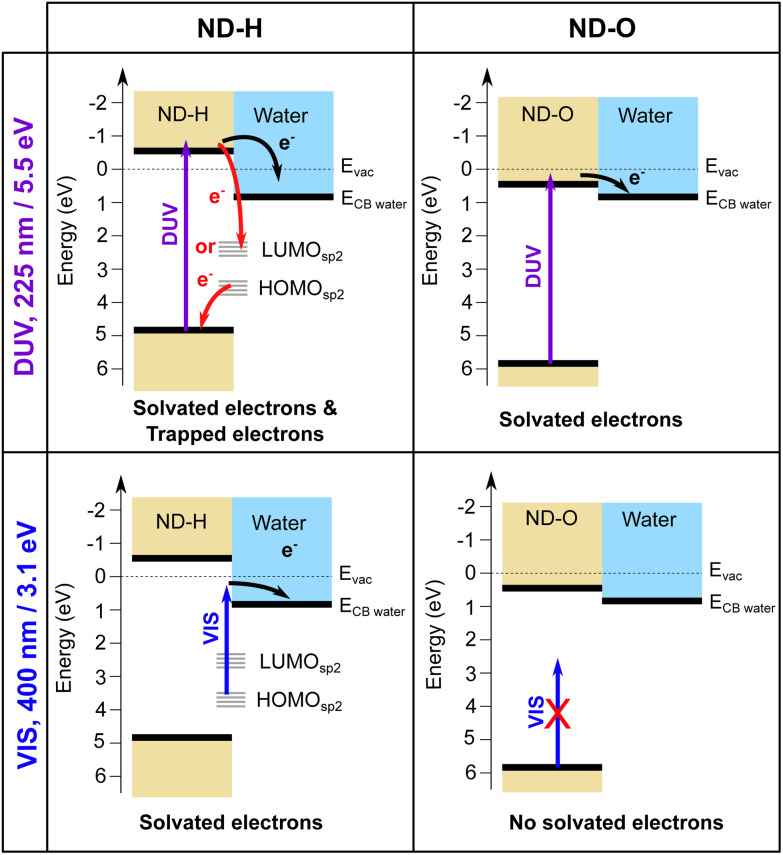
Schematic representation of the solvated electron emission process on ND-H and ND-O under DUV and VIS light excitation. ND-O refers to either ND-OH or ND-COOH for simplification. The HOMO and LUMO of sp^2^-hybridized surface states on ND-H are indicated in grey.

We interpret the different behaviour of ND-H compared to ND-OH and ND-COOH (further collectively referred to as ND-O) for both DUV and VIS excitation by distinct electron emission processes summarized in Fig. 3. First of all, surface termination affects the diamond electron affinity (EA).^8^ The EA of (111) diamond surfaces terminated with hydrogenated and hydroxylated surfaces were calculated in a vacuum and with a thin water film using DFT calculations (see the ESI†). While both surfaces were found to have a negative EA in a vacuum (−1.0 eV for H-termination and −0.4 eV for OH-termination), only the H-terminated surface maintains a negative EA with a thin adsorbed water film at neutral pH (−0.3 eV for H-termination and +0.7 eV for OH-termination). A positive EA is also expected for the carboxylated diamond surface.

Despite its positive EA, the electron emission is possible for ND-O because the water CBM might sit below the CBM of ND-O, as illustrated in Fig. 3. The surface termination of NDs strongly influences the orientation of the first water solvation shells *via* the strength of the hydrogen bonds.^32^ This can in turn modify the structure of the next few hydration layers and hence the EA of this interfacial water region around the ND. Previous DFT calculations have indeed shown that the bulk EA of water (0.2 eV) is different from surface water close to a vacuum interface (0.8 eV).^33^ In any case, the electron emission from hydrogen-terminated diamond is more efficient due to the larger energy difference between the diamond and water CBM, ensured by a negative EA, and the possibility to emit electrons through surface C–H states. Nevertheless, the H-terminated surface alone cannot explain electron emission upon VIS excitation, as it would only allow electron emission for excitation with photon energies above 4.5 eV.^13^ A different absorption process must therefore take place on ND-H upon VIS excitation.

In contrast, the negative ultrafast TA signal only observed for ND-H under DUV illumination suggests that an additional process occurs for ND-H during the first picoseconds. It could be due to either stimulated emission or ground-state bleaching of a species yet to be identified. Raman spectroscopy showed that the surface termination of ND-H is not only composed of CH_*x*_ groups but also contains small sp^2^-hybridization islands, as previously reported on similarly prepared ND-H.^34^ sp^2^ carbon quantification based on near-edge X-ray absorption fine structures (NEXAFS) on NDs hydrogenated, either by plasma or by annealing treatments, has concluded that up to 20% of the carbon atoms may be sp^2^-hybridized, predominantly those situated at the ND surface.^35^ Such sp^2^-hybridized carbon atoms are mostly localized on fullerene-like reconstructions (FLRs) which were also identified on annealed NDs.^36^ These non-diamond carbon atoms induce new states below the CBM of ND-H, which can contribute to visible light absorption and were found to increase significantly the photoluminescence of ND-H, compared to oxidized surfaces.^35,37^ To quantify the energy levels of an sp^2^-decorated ND, we performed hybrid-DFT calculations on a series of ND structures with various degrees of FLR coverage. The structures were optimized by tight-binding DFT and subsequent single point calculations were performed at the PBE0-D3/SVP level of theory (see section 4 and the ESI† for details). The corresponding structures and their orbital energies are depicted in Fig. 3.

**Fig. 4:**
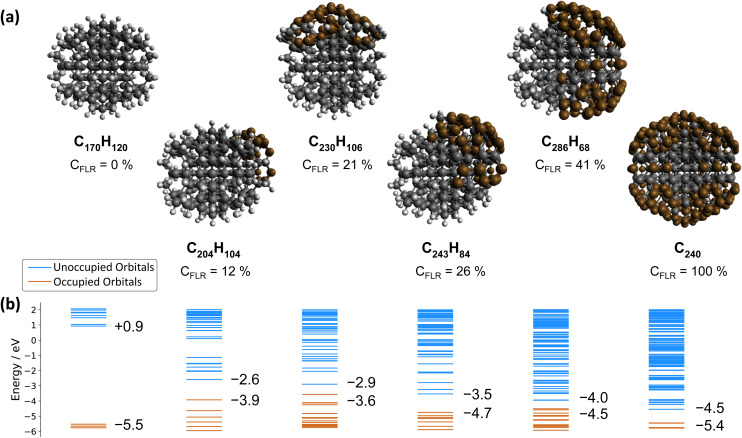
(a) Structures, sum formulae and FLR coverage (*C*_FLR_, at %) of the ND series. (b) Energies of the occupied and unoccupied orbitals. The HOMO and LUMO energies are given. From left to right: ND-H (C_170_H_120_), sp^2^-decorated NDs (C_240_H_104_, C_230_H_106_, C_243_H_84_, C_286_H_68_), and fully surface graphitized ND (C_450_). The sp^3^ carbon atoms are depicted in black, sp^3−*x*^ (*x* > 0) carbon atoms in brown, and hydrogen atoms in light grey.

ND-H has a negative electron affinity of −0.93 eV, which is similar to the calculation performed on H-terminated (111) diamond, and a HOMO–LUMO gap (HLG) of 6.46 eV. This HLG is higher than the bandgap of bulk diamond (5.5 eV) due to the quantum confinement effect of small-sized NDs.^38^ Upon addition of sp^2^ carbon on the ND, the frontier orbital energies are shifted upwards (HOMO) and downwards (LUMO), resulting in decreased HLGs (Fig. 4(b)). These frontier orbitals and the next few highest occupied/lowest unoccupied orbitals of the NDs partially covered by FLRs are all located in the respective sp^2^-hybridized carbon areas. Contour plots of the relevant orbitals are shown in the ESI.† With increasing FLR coverage, additional orbitals appear within the range of the ND-H's HLG. Interestingly, the HOMO energies are highest with a low amount of FLRs present and decrease with larger reconstructed areas. This decrease of HOMO energies is, however, not monotonic. In contrast, the systems’ LUMO energies decrease monotonically with increasing FLR coverage. We further computed the optical absorption spectra of smaller NDs with 0, 50 and 100 at% surface reconstruction, each surrounded by an explicit layer of water molecules (ESI†). The spectra confirm that the optical gap of a H-terminated ND is also significantly reduced upon surface reconstruction.

These findings support the hypothesis that sub-bandgap states are introduced in NDs upon formation of FLRs on their surface, and even a small coverage (*e.g.*, 12% of surface atoms) is sufficient to introduce them. The additional unoccupied surface states may act as trap states that, upon DUV excitation, induce ultrafast recombination of the excited electrons, which is detrimental to the solvated electron emission as illustrated in Fig. 3. An alternative process may be recombination of electrons from an occupied surface state with valence band holes in the excited ND. In either case, the electrons emerging from fully H-terminated areas on the ND-H surface may not be affected by these defect states and are then emitted in the water. As a result, the ultrafast TA response of the ND-H dispersion upon DUV excitation is associated with two parallel processes: (i) a strong recombination at early stages due to defect states and (ii) the emission of solvated electrons from fully hydrogenated regions on ND-H. The presence of sp^2^-hybridized defect states may explain the limited CO_2_ reduction activity of detonation hydrogenated NDs under DUV light compared to hydrogenated NDs with a higher crystallinity.^14^ In the case of ND-O, the amount of surface sp^2^ carbon is significantly reduced in the process of surface termination and therefore no such recombination is evidenced. Further TA investigations on the ns–μs timescale would be necessary to assess the recombination dynamics of the solvated electron with the ND surface after its emission, and to address the accompanying hole dynamics. Such experiments are, however, beyond the scope of the present study.

The emission of solvated electrons from ND-H upon VIS excitation can be explained by a combination of sp^2^-hybridized islands and fully H-terminated surface areas. FLRs may enable light absorption while hydrogen-terminated functional groups offer favourable negative EA properties. Such an inhomogeneous electron emission model was first proposed by Cui *et al.* to explain sub-bandgap electron emission from diamond surfaces in a vacuum.^20^ Our work demonstrates that a similar process occurs on ND with much smaller sp^2^-hybridized surface structures, which are stabilised by curvature effects not observed on larger diamond crystallites. We anticipate that by controlling more precisely the ratio between the hydrogenated surface groups and the FLR content, the electron emission properties of ND-H under VIS light excitation could be further optimized. Adding small graphitic channels in hydrogenated single crystal diamond may also lead to similar synergistic effects for electron emission induced by visible light.^39^ While sp^2^-hybridized states function as intermediate states absorbing in the VIS region, other (mixed) covalent surface functionalisation may be considered.^40^ An essential aspect for efficient solvated electron emission would be to ensure a good electron transfer between the absorbing surface states/molecules and the electron-emitting surface groups to benefit most from the unique electronic properties of H-terminated diamond surfaces. Future electron dynamics simulations, including the visualization of the electronic density migration,^41^ may support the understanding of the process and the design of functionality.

## 3 Conclusions

In summary, the early stages of electron emission in water from NDs upon illumination with DUV and VIS light were probed using ultrafast TA spectroscopy. While the emission of solvated electrons was observed for hydrogenated, hydroxylated and carboxylated NDs upon DUV illumination, it was only detected for hydrogenated NDs upon VIS excitation. This is interpreted by synergistic effects between sp^2^-hybridized carbon surface reconstructions, which serve as a pool of electrons to be excited with VIS light, and hydrogenated surface groups offering a local negative electron affinity facilitating electron emission. On the other hand, these FLRs are detrimental to electron emission upon DUV illumination as they behave as trap states increasing electron recombination. These findings may suggest that mixed ND surface chemistry composed of hydrogenated surface groups for electron emission and visible-light absorbing states—sp^2^-hybridized surface states in this work—should be considered for improving the electron emission properties of NDs and nanostructured diamond under solar excitation.

## 4 Methods

### 4.1 Materials

Nanodiamonds with controlled surface termination were used in this study. For ND-COOH, commercially available carboxylated detonation NDs (from Carbodeon, Vantaa and International Technology Center, USA) were dispersed in deionised (DI) water at a concentration of 10 mg mL^−1^ using an ultrasonic bath. For the preparation of ND-OH, 350 mg of detonation ND (Gansu Lingyun Corp.) were first milled in an attrition mill according to a literature procedure,^42^ and then dispersed in *ca.* 50 mL of deionized water and treated for 15 min in an ice-cooled ultrasonic bath. Then, Fe(ii)sulfate heptahydrate (14.6 g) was added and the mixture was dispersed for another 15 min. Subsequently, 7 mL of conc. sulfuric acid and 7 mL of H_2_O_2_ solution (30%) were added and the mixture was sonicated for ≈90 min while keeping the temperature at 0 °C. 130 mL of DI water were added to stop the reaction and the hydroxylated diamond was precipitated and washed through consecutive centrifugation/washing cycles with water (3×), 3 M HCl (3×), acetone (3×), and again water (3×), until neutral pH was reached and the inorganic components were removed. The material was centrifuged and dried *in vacuo*. For the reduction of residual carbonyl groups, borane reduction was performed as the second step. The dried NDs treated with the Fenton reagent were dispersed first in dry tetrahydrofuran (THF), then 15 mL of 1 M borane solution in dry THF was added dropwise and then the reaction mixture was stirred for 5 days under a nitrogen atmosphere. After addition of 30 mL of 3 M HCl the product ND-OH was purified by consecutive centrifugation–washing cycles with water (3×), acetonitrile (3×), acetone (3×), isopropanol (3×) and again water (3×). The particles can be isolated by centrifugation and subsequent drying *in vacuo*. Hydrogenation of NDs, yielding ND-H, was realized by H_2_ plasma treatment using the same milled detonation ND as for ND-OH.^43^ 30 mg of the initial ND were placed in a quartz tube and exposed to a H_2_ microwave plasma with a gas pressure of 12 mbar, a gas flow of 10 standard cubic centimetres per minute and a microwave power of 250 W, for 20 min. ND-H was then dispersed in ultrapure water (18.2 MΩ cm) by ultrasonication (Hielscher UP400s, 300 W, 24 kHz) for 1 h under cooling, and centrifuged (40 min, 2400 g) to remove aggregates. The final concentration was calculated by measuring the mass of residue after drying a calibrated volume of the initial suspension.

### 4.2 Ultrafast transient absorption

Transient absorption (TA) spectroscopy was performed using a custom-built setup in standard transmission geometry with near-vertical incidence of pump and probe light. The setup used was pumped using a commercial Ti:sapphire laser system (Coherent Inc., Legend Elite Duo), delivering pulses of 25 fs duration at 800 nm central wavelength and 2.5 mJ pulse energy with a repetition rate of 5 kHz.^44^

#### 4.2.1 Pump-light generation

Visible excitation light centred at 400 nm (3.1 eV) was generated by frequency doubling about 0.2 mJ of the fundamental pulse in a β-BaB_2_O_4_ (BBO) crystal. Deep-UV (DUV) pump pulses centred at 225 nm (5.5 eV) were generated by sum-frequency mixing of 400 nm and 514 nm pulses in BBO, respectively. The latter were obtained using the output of an optical-parametric amplifier (OPA, Coherent Inc., OperA Solo). The maximum energy of the pump beam at the sample position was about 0.2 μJ per pulse for the DUV light, and about 8 μJ for the visible light. In each case, the excitation spot had a 1/*e*^2^ diameter of about 600 μm, resulting in a radiant exposure of about 70 μJ cm^−2^ (DUV) and 2.8 mJ cm^−2^ (VIS), respectively. Keeping in mind the respective photon energies of the VIS and DUV pulses, the actual photon number density per unit area is about 70 times higher under visible light excitation.

#### 4.2.2 Probe-light generation

The transient absorption changes were probed over the visible and near-infrared spectral range with a white light continuum (WLC). Depending on the conditions of pump generation, two WLC generation schemes were used. When VIS pump light was used, IR pulses centred at 1300 nm, generated by the OPA, were used for supercontinuum generation in a 3 mm thick c-cut sapphire substrate. Thereby, the WLC spans the whole VIS/NIR probing range without being obstructed by the WLC pump light. Under DUV pump conditions, the fundamental wavelength at 800 nm was instead used for WLC generation, since the OPA was blocked by the DUV generation scheme. This restricts the effective probe range to wavelengths below about 760 nm. Although the probe pulses were not further polarized in these experiments, the WLC had predominantly the same linear polarization as its respective pump pulse. The probe spot had a 1/*e*^2^ diameter of about 200 μm, and the probe radiant exposure was typically below 1 nJ cm^−2^ nm^−1^.

#### 4.2.3 Data acquisition

After passing through the sample, the probe pulse was spectrally dispersed using a Czerny–Turner spectrograph (Andor Technology, Shamrock 303) and detected with a multichannel detector (Andor Technology, Newton DU920P-BEX2-DD). Transient spectra were recorded up to a pump–probe delay of 30 ps using an optical delay stage. A semi-logarithmic delay scheme was used, providing a dense linear sampling during the ultrafast period, and logarithmic sampling afterwards.^45^ The maximum detector read-out speed was about 200 Hz, limiting the number of integrated pump–probe spectra per delay time to a few hundreds.

### 4.3 Theoretical methods

For the computation of the orbital energies of NDs with different amounts of sp^2^-hybridized surface coverage, structures were optimized by tight-binding density functional theory (DFTB) using xtb software^46^ and default optimization conditions. This yields reasonable structures, as discussed in the ESI.† On these structures, we performed single point calculations within ORCA 5.0,^47^ using the PBE0 hybrid functional,^48^ Ahlrich's def2-SVP basis set,^49^ Grimme's third order dispersion correction (D3) with Becke–Johnson damping,^50^ and the ORCA's DEFGRID1 option for fast integral calculations. The base structures used for the calculations of orbital energies and absorption spectra were obtained from the CSIRO twinned nanodiamond data set.^51^ Most computations were carried out on the high performance computing cluster CURTA at the Zentraleinrichtung Datenverarbeitung (ZEDAT) of Freie Universität Berlin.^52^

## Author contributions

FB: data curation, investigation, validation, visualization, and writing – review & editing. TK: software, validation, visualization, and writing – review & editing. AV: investigation. HG: investigation. JCA: conceptualization, funding acquisition, project administration, and resources. BK: investigation. AK: conceptualization, funding acquisition, project administration, resources, supervision, and writing – review & editing. KL: investigation, funding acquisition, project administration, and writing – review & editing. AB: conceptualization, investigation, software, supervision, validation, and writing – review & editing. TP: conceptualization, funding acquisition, investigation, project administration, supervision, visualization, and writing – original draft. CM: conceptualization, data curation, investigation, resources, validation, visualization, and writing – original draft.

## Conflicts of interest

There are no conflicts to declare.

## Supplementary Material

NR-014-D2NR03919B-s001
