# Supplementary material for: Early dynamics of the emission of solvated electrons from nanodiamonds in water
Source: Nanoscale. 2022 Nov 1;14(46):17188–95. doi: 10.1039/d2nr03919b (PMC9714771; doi:10.1039/d2nr03919b)
Supplement: NR-014-D2NR03919B-s001 [file NR-014-D2NR03919B-s001.pdf]

Electronic Supplementary Material (ESI) for Nanoscale.
This journal is © The Royal Society of Chemistry 2022

Electronic Supplementary Information: Early dynamics of the emission of solvated electrons from nanodiamonds in water

Franziska Buchner[a,b], Thorren Kirschbaum[a,c], Amélie Venerosy[d], Hugues Girard[d,e], Jean-Charles Arnault[d,e], Benjamin Kiendl[b], Anke Krueger[b,f], Karin Larsson[g], Annika Bande[a], Tristan Petit[a], and Christoph Merschjann[a]

[a] *Helmholtz-Zentrum Berlin für Materialien und Energie GmbH, Hahn-Meitner-Platz 1, 14109 Berlin, Germany*

[b] *Institut für Organische Chemie, Julius-Maximilians-Universität Würzburg, Am Hubland, D-97074 Würzburg, Germany*

[c] *Freie Universität Berlin, FB Mathematik & Informatik, Artificial Intelligence for the Sciences, Arnimallee 12, D-14195 Berlin, Germany*

[d] *CEA,LIST, Diamond Sensors Laboratory, Bâtiment 451, PC 45, 91191 Gif sur Yvette Cedex, France*

[e] *Université Paris-Saclay, CEA, CNRS, NIMBE, 91191 Gif sur Yvette Cedex, France*

[f] *Institute for Organic Chemistry, Stuttgart University, Pfaffenwaldring 55, 70569 Stuttgart, Germany* and

[g] *Uppsala University, Lägerhyddsvägen 1, 751 21, Uppsala, Sweden* *

(Dated: July 17, 2022)

I. TRANSIENT ABSORPTION: DATA TREATMENT

Transient absorption data are subject to a wavelength-dependent time of pump-probe interaction, caused by the chirp of the broadband probe light [1]. This chirp was manually modelled using a polynomial of degree 2 on the photon-energy scale, and the data were thus corrected for time-zero dispersion [1]. Typically, resonant TA signals are accompanied by non-resonant cross-phase-modulation signals (XPM, also known as "coherent artefact"), caused by two-photon interaction between pump and probe light pulses [1, 2]. This XPM is very prominent in the case of visible pump (Figure S1), showing three components, one for the two cuvette windows, and one for the solvent, respectively. A similar behaviour was observed for the NDs and NaI solutions. The fact that we did not observe sizeable XPM in the DUV-pumped data is likely due to the comparably low photon number density of the DUV pulses. Considering the radiant fluxes, we can expect the

* christoph.merschjann@helmholtz-berlin.de; tristan.petit@helmholtz-berlin.de

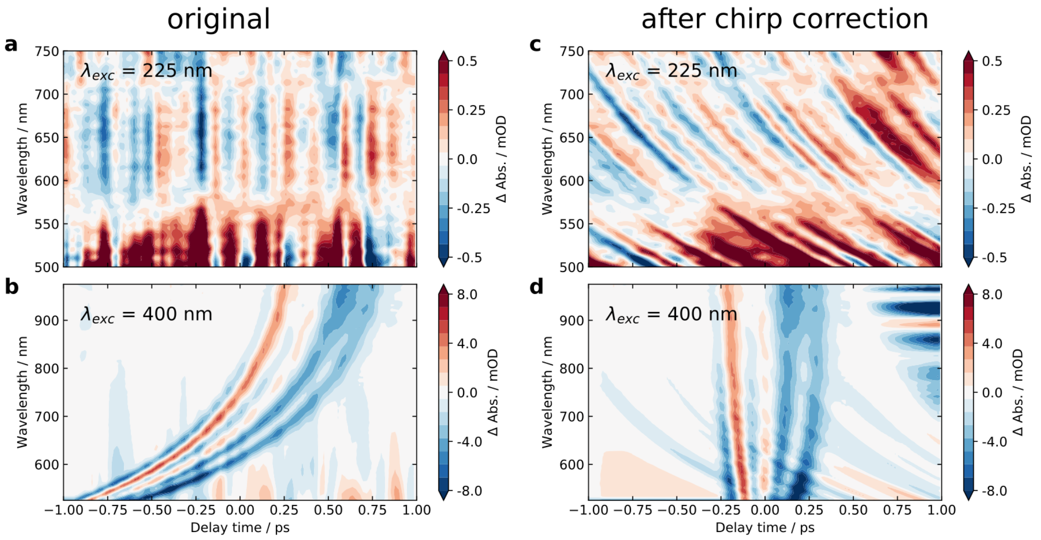

FIG. S1. TA signal from pure H_2O as a function of probe wavelength and delay time for DUV (a) and visible (b) excitation. The signal in b is due to the coherent artefact (XPM), caused by the pump-probe interaction in two cuvette windows and the fluid inside the cuvette. Remarkably, this signal is absent in a, probably due to about 70 times lower photon number density. Panels c and d show the signals after correcting for the chirp, respectively. In d, the second XPM feature has been chosen as time-zero reference.

XPM in the latter case to be below the noise level. The right panels show the same data after chirp correction. Note that in case of VIS excitation, the second XPM feature has been chosen as time-zero reference point, as it represents the interaction with the liquid phase in the cuvette. The first and third are due to the front and back cuvette windows, respectively.

We attempted to perform a global fit of the 2D TA map. Several sets of converging fitting parameters were obtained for different initial conditions, showing that the fitting was not robust. We attribute this to the lack of spectral features in the TA response. As a result, the discussion was restricted to qualitative description in the main article.

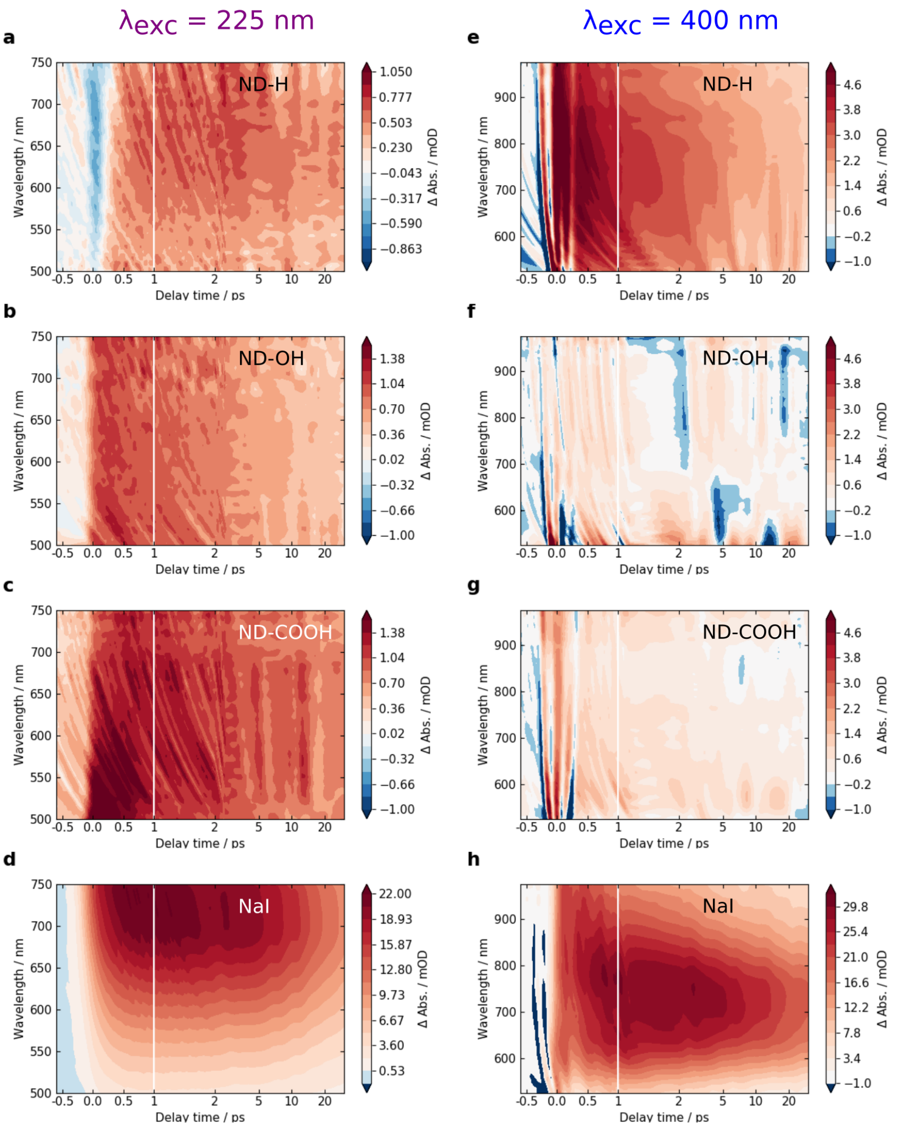

FIG. S2. TA signal from ND-H, ND-OH, ND-COOH and NaI samples as a function of probe wavelength and delay time for DUV (a-d) and visible (e-h) excitation, respectively.

II. TRANSIENT ABSORPTION 2D MAPS OF NANODIAMONDS

Figure S2 shows an overview of the TA 2D maps for the samples used in this work, both for 225 nm and 400 nm excitation. The respective TA spectra, averaged between 1 and 5 ps for both DUV and VIS excitation in all samples, are shown in Figure S3. Additionally, Figure S4 presents a comparison of two ND-COOH samples obtained from different sources. As can be seen, the general trend of the TA signals is maintained, indicating that the TA behaviour is mainly driven by the surface termination.

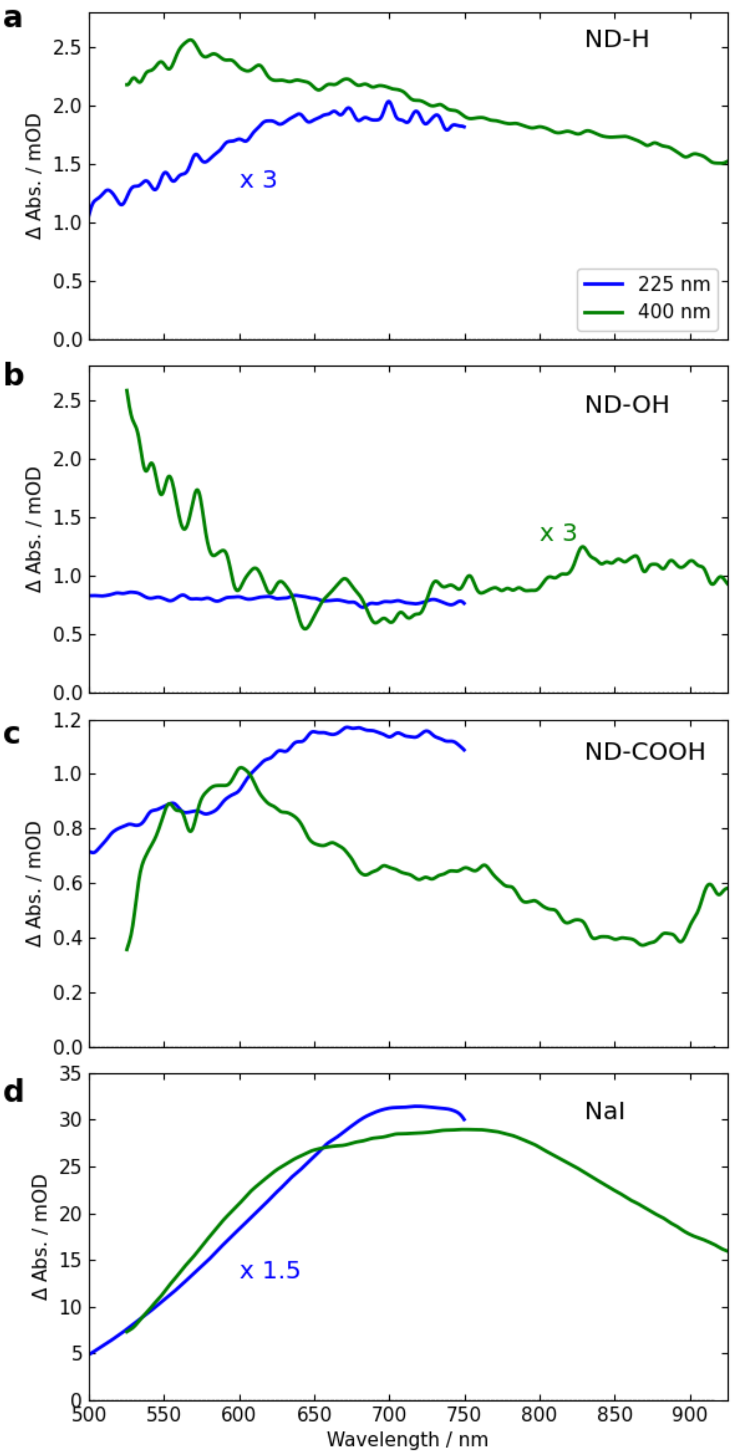

FIG. S3. TA spectra from ND-H, ND-OH, ND-COOH and NaI samples as a function of probe wavelength for DUV (blue) and VIS (green), respectively. The spectra were obtained by averaging the results shown in Figure S2 between 1 and 5 ps.

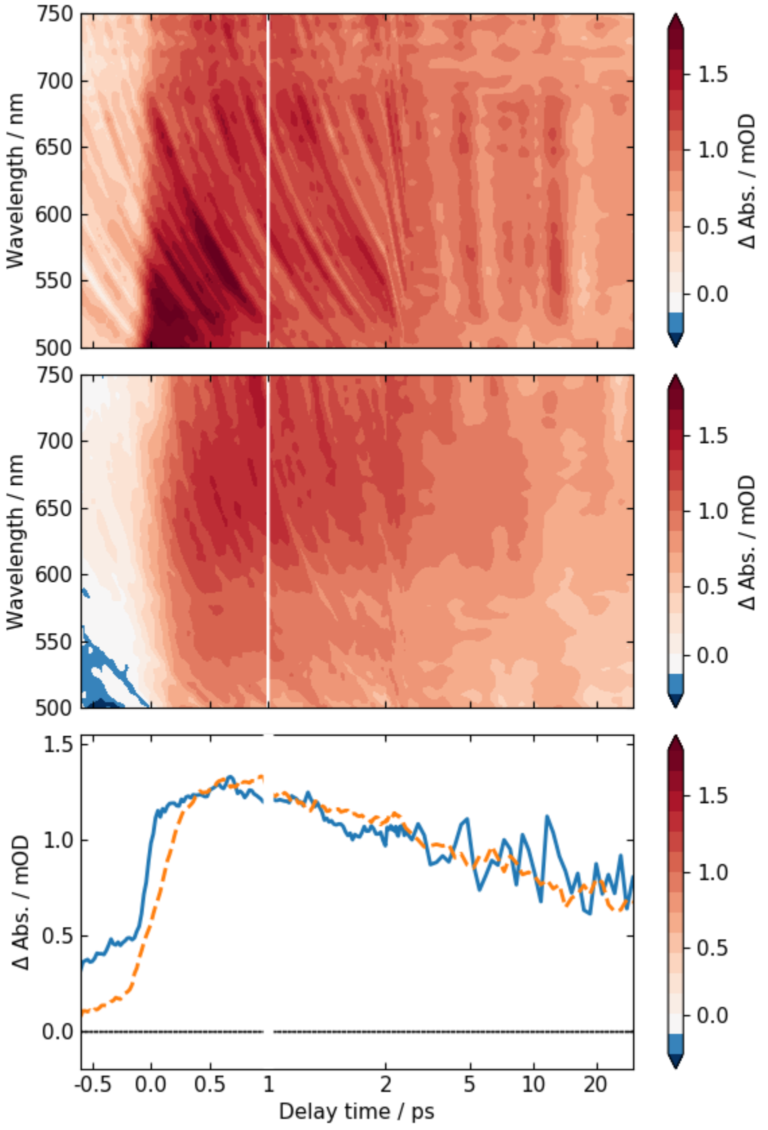

FIG. S4. TA signal from ND-COOH samples obtained from detonation (ITC, 5 nm average diameter) and high-pressure high-temperature (Syndia, 20 nm average diameter) synthesis under DUV excitation. The lowest panel shows the comparison of TA traces integrated between 600 nm and 740 nm.

III. ELECTRON AFFINITY CALCULATION AT THE (111) DIAMOND-WATER INTERFACE

DFT calculations under periodic boundary conditions were used to model the electronic properties of a diamond (111) surface with H-/OH-termination. More specifically, an ultrasoft pseudopotential [3] plane-wave approach was used, based on the PBE [4] generalized gradient approximation

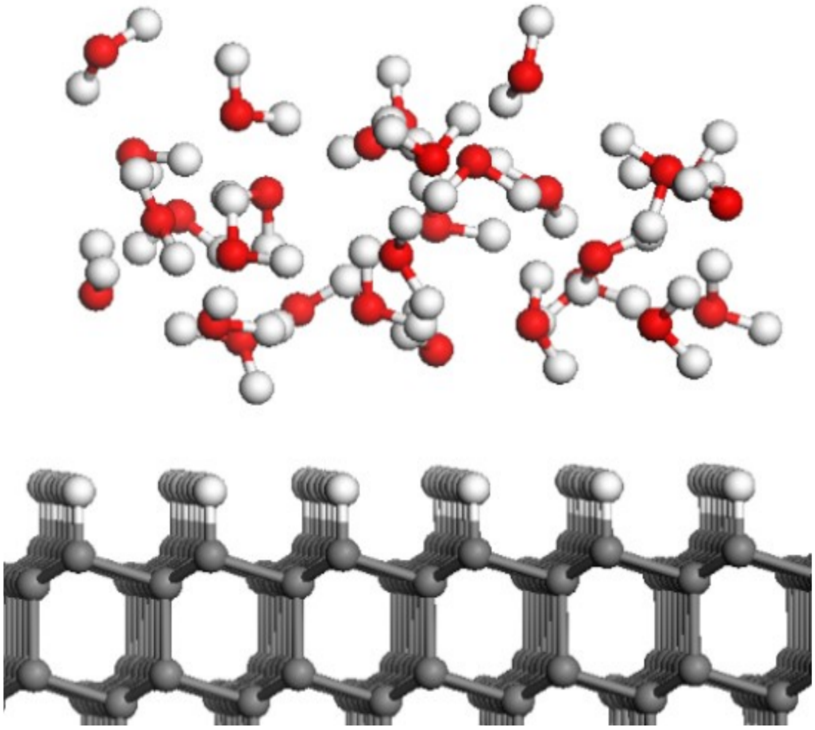

FIG. S5. Elemental cell of the model of a neutral very thin water adlayer onto an H-terminated non-doped diamond (111) surface.

(GGA) of the exchange-correlation functional. Due to the presence of unpaired electrons in the systems, all of the calculations were based on spin-polarized GGA (i.e., GGSA). With a few exceptions, the calculations of this part of the study were carried out using the Cambridge Sequential Total Energy Package (CASTEP) program from BIOVIA, Inc. [5]. To evaluate the accuracy of these calculations, the calculated band gap for bulk diamond was compared with the experimental one. As a result, very similar values were obtained: 5.40 eV vs. 5.47 eV. This approach has already been proven to be successful in modelling electron transfer from diamond (100) surfaces to various aqueous adsorbates [6, 7]. Two terminated undoped diamond (111) surfaces were investigated, based upon the terminating species H and OH. In addition, a thin neutral water adlayer was attached to these diamond surfaces. An example unit cell is shown in Figure S5, the electron affinities are summarized in Table S1.

A terminated diamond (111) surface that is in contact with a water adlayer shows a completely different picture than a surface in vacuum. In the presence of water, additional states are formed in the band gap of the surface for the H- and OH- termination scenarios. The surface C atoms, H atoms, and the water adlayer all contribute to the major part of these band gap states. This demonstrates that thin water adlayers will chemically interact with the diamond surfaces.

TABLE S1. Electron affinity (EA) for (111) diamond with H/OH-terminations and vacuum/water adlayers.

	EA in vacuum (eV)	EA in water (eV)
Diamond-H	−1.0	−0.3
Diamond-OH	−0.4	+0.7

IV. VALIDITY OF XTB GEOMETRY OPTIMIZATION FOR NDS

We here discuss the quality of the methods employed for the calculation of ND orbital energies. We used the fast and robust tight-binding based xtb program package for optimizing the ND structures and subsequently computed orbital energies at PBE0-D3-SVP level of theory ("Fast Method"). We compare the frontier orbital energies obtained in this way for three small model systems to the fully PBE0-D3-TZVP optimized structures ("Reference Method") [8] in Table S2. The model systems are two small fully hydrogenated NDs ($C_{35}H_{36}$, $C_{53}H_{48}$) and a small ND that is fully covered by amorphous carbon (C_{88}). Note that the use of more accurate methods for structure optimization or single-point calculation was not computationally feasible for the structures presented in the main paper. The frontier orbital energies computed by the fast method are qualitatively similar to those computed by the reference method. The dispersed LUMOs of the H-terminated NDs are better described by the larger basis set of the reference method, hence, the LUMO energy and HOMO-LUMO gap obtained from the fast method are overestimated by about 0.5 eV. In contrast, the LUMO energy of C_{88} is underestimated by about 0.4 eV by the fast method, resulting also in an underestimation of its HOMO-LUMO gap. The HOMO energies of all systems are relatively well described by the fast method (absolute deviations < 0.15 eV). Despite the introduction of errors, the fast method yields frontier orbital energies that are in qualitative agreement with the reference method. Thus, the fast method can be trusted for the use of qualitative comparisons, as is done in the main paper.

V. ORBITAL SHAPES OF SURFACE-RECONSTRUCTED NDS

Here we present and discuss the orbital shapes of the NDs series with different level of surface graphitization for which the orbital energies were discussed in the main paper. H-terminated NDs have uniquely shaped frontier orbitals, in particular, their LUMOs are highly delocalized over the surface of the ND. Upon addition of a sp^2-hybridized surface reconstructions on ND-H, the frontier orbitals are instead located on the reconstructed patch. However, in the higher unoccupied orbitals

TABLE S2. HOMO, LUMO and HOMO-LUMO-Gap (HLG) energies for $C_{35}H_{36}$, $C_{53}H_{48}$ and C_{88}, computed at different levels of theory for geometry optimization and single-point calculation. "Fast Method" means an XTB optimization and PBE0-D3-SVP single point calculation, and "Reference Method" a PBE0-D3-TZVP optimization and single-point calculation. All energies are given in eV.

Method	Geometry Opt.	Single Point	E(HOMO)	E(LUMO)	E(HLG)
$C_{35}H_{36}$					
Fast Method	XTB	PBE0-D3-SVP	−6.688	1.084	7.773
Reference Method	PBE0-D3-TZVP	PBE0-D3-TZVP	−6.626	0.518	7.144
$C_{53}H_{48}$					
Fast Method	XTB	PBE0-D3-SVP	−6.203	1.107	7.310
Reference Method	PBE0-D3-TZVP	PBE0-D3-TZVP	−6.177	0.515	6.692
C_{88}					
Fast Method	XTB	PBE0-D3-SVP	−5.583	−4.771	0.812
Reference Method	PBE0-D3-TZVP	PBE0-D3-TZVP	−5.720	−4.342	1.379

of these structures we find spread-out orbitals on the H-terminated surface that resemble the pure ND-H's LUMO. These orbitals are shown in Figure S6, together with the molecular structures, sum formulae and percentage of reconstructed carbon coverage. With increasing amount of surface reconstruction, more virtual orbitals are present between the LUMO and the ND-H-LUMO-like virtual orbital, and their energies decrease on trend. This trend is, however, not monotonous as it may also depend on the structure's specific geometrics and symmetry. This implies that while sp^2-hybridized carbon surface atoms modify the particles' electronic structures by adding additional states in their optical gaps, the original electronic structure of hydrogen-terminated NDs is partly retained.

VI. OPTICAL ABSORPTION OF RECONSTRUCTED NDS FROM DFT

For the computation of absorption spectra, structures were DFT-optimized using the revised PBE (revPBE) functional [9] and Ahlrich's def2-SVP basis set [10]. The fully reconstructed ND structure contains a core of 15 diamond-like and a shell of 70 sp^2-hybridized carbon atoms, the half-reconstructed ND has 30 of the sp^2-carbons removed and the remaining structure saturated by hydrogen atoms, and the last structure is a Td-symmetric ND-H with sum formula $C_{35}H_{36}$. We also calculated the corresponding spectra for a larger ND-H with 66 C atoms and found them to be similar to those of the smaller ND-H. Absorption spectra were calculated by time-dependent DFT

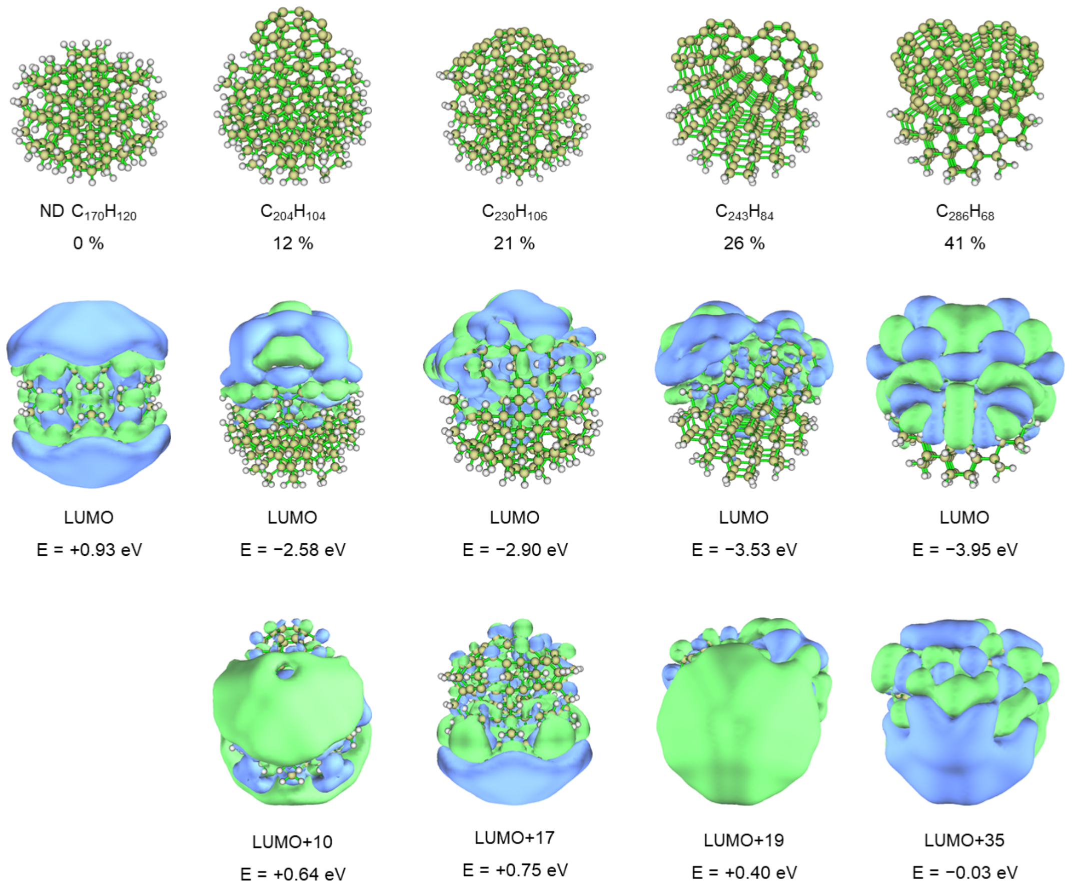

FIG. S6. Orbital shapes of the ND-H/reconstructed ND structure series. Top row: Structures, sum formula, sp^{3-x} ($x > 0$) carbon coverage in at%. Middle row: LUMO shapes and energies. Bottom row: LUMO+n shapes and energies of the lowest unoccupied orbital that resembles the ND-H's LUMO. C atoms in yellow, H atoms in light grey, bonds in green, orbital contour plots of isovalue 0.002.

in the Tamm-Dancoff approximation using the PBE0 hybrid functional [11] and the same def2-SVP basis set and normalized to (0,1) intensity. Here, the RIJCOSX approximation [12] and the appropriate auxiliary basis set [13] were used to speed up integral calculations, and dispersion correction was included through Grimme's third order atom-pairwise dispersion correction with Becke-Jones damping [14]. The spectra were calculated using the lowest 15 excited states of each system. These calculations were performed within ORCA, version 4.2.1 [12]. For the computation of spectra, approximately one monolayer of water molecules is placed explicitly around each structure to account for the effects of water solvation. During the optimization of the fully sp^2-covered ND in water, one water molecule splits to form a hydroxyl group at the graphitized surface area, leaving one proton in the water layer. This effect was observed for three independent optimization runs starting at different initial configurations, indicating a strong oxygen affinity for this structure, as previously

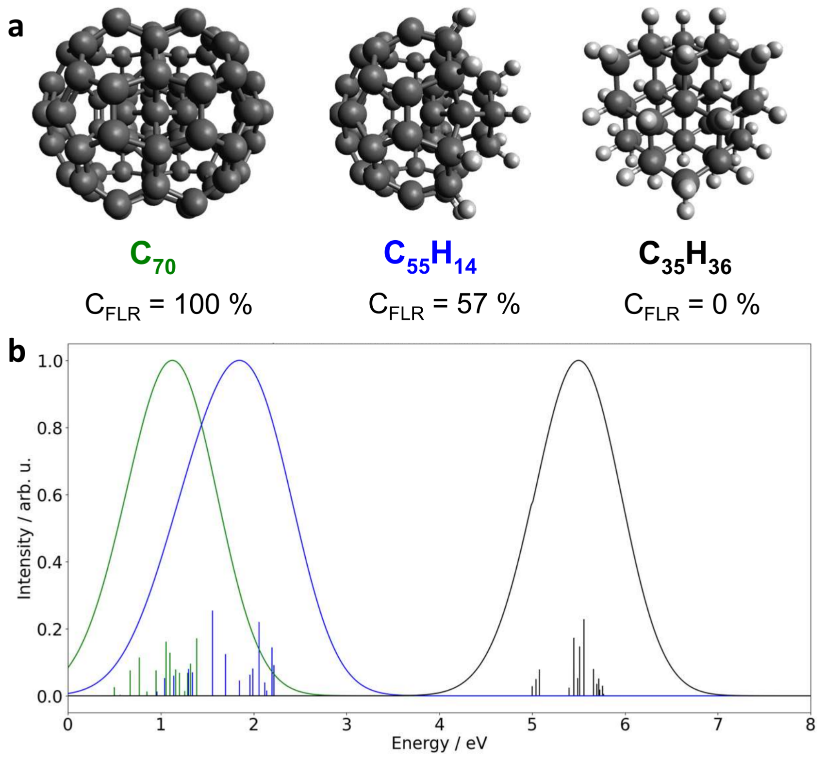

FIG. S7. Effect of nanodiamond graphitization on optical absorption. (a) Calculated structures for nanodiamonds with 0, 50 and 100 at% surface coverage of sp^{3-x} ($x > 0$) carbon. The explicit layer of water molecules used for the calculation is not shown for clarity. (b) DFT calculation of optical absorption bands for the structures shown in a with a water monolayer coverage. Note that only the first 15 excited states are calculated each.

experimentally evidenced [15]. The ND/water absorption spectrum was calculated for this singly hydroxylated structure. The absorption spectra of a fully sp^2-covered small ND, a half-covered ND and a fully hydrogenated ND are shown in Figure S7. While a fully hydrogen-terminated ND only enables light absorption above 5 eV in water, the light absorption onset shifts progressively to lower energies based on the degree of surface reconstruction. Note that considering the small size of the calculated diamond cluster relative to detonation NDs, a quantitative comparison of the absorption energies appears difficult. However, these results are in qualitative agreement with the optical gaps computed for sp^2-covered NDs-H (see main text) and experimental absorption spectra of ND-H presenting a long absorption tail up to ≈ 600 nm (2 eV) [16]. Note that computation of spectra of much larger systems is not feasible with the computational resources at hand.

[1] U. Megerle, I. Pugliesi, C. Schriever, C. F. Sailer, and E. Riedle, Sub-50 fs broadband absorption spectroscopy with tunable excitation: Putting the analysis of ultrafast molecular dynamics on solid ground, Appl. Phys. B **96**, 215 (2009).

[2] S. A. Kovalenko, A. L. Dobryakov, J. Ruthmann, and N. P. Ernsting, Femtosecond spectroscopy of condensed phases with chirped supercontinuum probing, Phys Rev A **59**, 2369 (1999).

[3] D. Petrini and K. Larsson, Origin of the reactivity on the nonterminated (100), (110), and (111) diamond surfaces: An electronic structure DFT study, J Phys Chem C **112**, 14367 (2008).

[4] J. P. Perdew, K. Burke, and M. Ernzerhof, Generalized gradient approximation made simple, Phys Rev Lett **77**, 3865 (1996).

[5] S. J. Clark, M. D. Segall, C. J. Pickard, P. J. Hasnip, M. I. J. Probert, K. Refson, and M. C. Payne, First principles methods using CASTEP, Z. Kristallogr. Cryst. Mater. **220**, 567 (2005).

[6] O. Manelli, S. Corni, and M. C. Righi, Water adsorption on native and hydrogenated diamond (001) surfaces, J Phys Chem C **114**, 7045 (2010).

[7] M. M. Hassan and K. Larsson, Effect of surface termination on diamond (100) surface electrochemistry, J Phys Chem C **118**, 22995 (2014).

[8] D. López-Carballeira and T. Polcar, Ab initio description of nanodiamonds: A DFT and TDDFT benchmark, Diam Relat Mater **108**, 107959 (2020).

[9] Y. Zhang and W. Yang, Comment on "generalized gradient approximation made simple", Phys Rev Lett **80**, 890 (1998).

[10] F. Weigend and R. Ahlrichs, Balanced basis sets of split valence, triple zeta valence and quadruple zeta valence quality for H to Rn: Design and assessment of accuracy, Phys Chem Chem Phys **7**, 3297 (2005).

[11] C. Adamo and V. Barone, Toward reliable density functional methods without adjustable parameters: The PBE0 model, J. Chem. Phys. **110**, 6158 (1999).

[12] F. Neese, F. Wennmohs, U. Becker, and C. Riplinger, The ORCA quantum chemistry program package, J. Chem. Phys. **152**, 224108 (2020).

[13] F. Weigend, Accurate coulomb-fitting basis sets for H to Rn, Phys Chem Chem Phys **8**, 1057 (2006).

[14] S. Grimme, S. Ehrlich, and L. Goerigk, Effect of the damping function in dispersion corrected density functional theory, J Comput Chem **32**, 1456 (2011).

[15] T. Petit, J.-C. Arnault, H. A. Girard, M. Sennour, T.-Y. Kang, C.-L. Cheng, and P. Bergonzo, Oxygen hole doping of nanodiamond., Nanoscale **4**, 6792 (2012).

[16] G. Thalassinos, A. Stacey, N. Dontschuk, B. J. Murdoch, E. Mayes, H. A. Girard, I. M. Abdullahi, L. Thomsen, A. Tadich, J.-C. Arnault, V. N. Mochalin, B. C. Gibson, and P. Reineck, Fluorescence and Physico-Chemical Properties of Hydrogenated Detonation Nanodiamonds, C **6**, 7 (2020).
